# A Survey of Noninteractive Zero Knowledge Proof System and Its Applications

**DOI:** 10.1155/2014/560484

**Published:** 2014-05-04

**Authors:** Huixin Wu, Feng Wang

**Affiliations:** ^1^Department of Information Engineering, North China University of Water Conservancy and Electric Power, Zhengzhou 450011, China; ^2^School of Software, North China University of Water Conservancy and Electric Power, Zhengzhou 450011, China

## Abstract

Zero knowledge proof system which has received extensive attention since it was proposed is an important branch of cryptography and computational complexity theory. Thereinto, noninteractive zero knowledge proof system contains only one message sent by the prover to the verifier. It is widely used in the construction of various types of cryptographic protocols and cryptographic algorithms because of its good privacy, authentication, and lower interactive complexity. This paper reviews and analyzes the basic principles of noninteractive zero knowledge proof system, and summarizes the research progress achieved by noninteractive zero knowledge proof system on the following aspects: the definition and related models of noninteractive zero knowledge proof system, noninteractive zero knowledge proof system of NP problems, noninteractive statistical and perfect zero knowledge, the connection between noninteractive zero knowledge proof system, interactive zero knowledge proof system, and zap, and the specific applications of noninteractive zero knowledge proof system. This paper also points out the future research directions.

## 1. Introduction

In 1985, Goldwasser et al. [1] first put forward the concept of interactive proof system and analyzed the interactive proof system whose knowledge complexity is zero, which created an important branch of cryptography and computational complexity theory—zero knowledge proof. The most attractive feature of zero knowledge proof lies in its seemingly contradictory unique nature that a prover can prove the correctness of an assertion to the verifier without leaking any extra information. It can force the malicious participants in cryptographic protocol to execute in accordance with predetermined steps to ensure the safety of the protocol. Thus it has a broad application prospect. To speak vividly, a verifier who receives the zero knowledge proof of a statement is supposed to be told by God that it is true. The main features of zero knowledge proof system include completeness, soundness, and zero knowledge.

*Completeness*. If the statement is correct, then the verifier will “always” accept.

*Soundness*. If the statement is incorrect, then the verifier will “always” reject.

*Zero Knowledge*. No (malicious) verifier can get any extra information from the proof procedure, except the correctness of the statement. 

Blum et al. [2, 3] first study the noninteractive zero knowledge (hereinafter referred to as NIZK) proof system and present the common reference string model that is generally applied at present. Noninteractive zero knowledge proof system contains only a message sent by a prover to verifier, which can be better used in the construction of cryptographic protocols. Thereafter, researches on the theory and applications of NIZK proof system have started successively, including NIZK proof of NP problems and noninteractive statistical (perfect) zero knowledge as well as the application of NIZK proof to CCA security encryption scheme, anonymous authentication, and the construction of group and ring signature.

In recent years, Groth et al. suggest to turn the research of NIZK to specific problems [4–17] and construct NIZK proof systems based on different application scenarios. This idea greatly improves the efficiency and practicability of NIZK and created a new line of research on the applications of NIZK. In the subsequent sections of this paper, we will elaborate the relevant concepts of NIZK proof and summarize the main research results of NIZK.

## 2. Preliminary Knowledge

In the following, let {0,1}^*n*^ denote the set of *n*-bit strings and let {0,1}* denote the set of all strings. Two probability ensembles are said to be computationally indistinguishable (denoted by ≈_*c*_), if no probabilistic polynomial time Turing machine can distinguish them with nonnegligible probability. Two probability ensembles are said to be statistically indistinguishable or statistically close (denoted by ≈_*s*_), if their statistical distance is negligible.

### 2.1. Zero Knowledge Interactive Proof System

**Definition 1 (zero knowledge interactive proof system)**

For language *L*⊆{0,1}* and a pair of interactive Turing machines (*P*, *V*), in which *P* possesses unlimited computational power and *V* is probabilistic polynomial time, (*P*, *V*) is said to be zero knowledge interactive proof system of language *L* if the following three conditions are true.

(1) Completeness: for any common input *x* ∈ *L* and polynomial *p*(·),
  (1) $$\begin{array}{c} \text{Pr}\left[\left(P,V\right)\left(x\right)=1\right]\geq 1-\frac{1}{p\left(\left|x\right|\right)}. \end{array}$$
(2) Soundness: for any common input *x* ∉ *L* and any interactive Turing machine *P*′ and polynomial *p*(·),
  (2) $$\begin{array}{c} \text{Pr}\left[\left(P^{\prime },V\right)\left(x\right)=1\right]<1-\frac{1}{p\left(\left|x\right|\right)}. \end{array}$$
(3) Zero knowledge: for each probabilistic polynomial time Turing machine V*, there is a probabilistic polynomial time algorithm M* such that, for any *x* ∈ *L*,
  (3) (P,V∗)(x)≈Mc∗(x).

*P* is called the prover and *V* is called the verifier.

Intuitively speaking, completeness reflects correctness of the system, which means, for valid input *x* ∈ *L*, a prover can always complete the proof successfully such that the verifier accepts. Soundness is defined against the malicious prover, which means, for invalid input *x* ∉ *L*, no prover *P*′ can construct a valid proof system such that the verifier accepts. While for the verifier, zero knowledge means no malicious verifier is able to derive extra knowledge from the process of interaction.

In addition, according to different computational capabilities of the prover and verifier, the above properties (2) and (3) can also be modified, respectively. If the indistinguishability of the two probability ensembles in property (3) is statistically indistinguishable or identically distributed, zero knowledge will be correspondingly defined as statistical zero knowledge and perfect zero knowledge. On the other hand, if soundness holds for any probabilistic polynomial time prover, that is, computational soundness, then the interactive proof system is called the zero knowledge argument system [18].

### 2.2. Noninteractive Zero Knowledge

Since it has been shown that, in the plain model, only languages in BPP have NIZK proof systems, therefore the definition for NIZK proof system usually contains an initial set-up assumption. At present, it is generally accepted by the researchers to construct NIZK proof system in the common reference string (hereinafter referred to as CRS) model.

**Definition 2 (NIZK proof system)**

For a pair of probabilistic Turing machines (*P*, *V*), in which *P* is probabilistic polynomial time and *V* is deterministic polynomial time, (*P*, *V*) is called the noninteractive zero knowledge proof system for language *L* if the following conditions are met.

(1) Completeness: for any common input *x* ∈ *L* and polynomial *p*(·),
  (4) $$\begin{array}{c} \text{Pr}\left[V\left(x,R,P\left(x,R\right)\right)=1\right]\geq 1-\frac{1}{p\left(\left|x\right|\right)}. \end{array}$$
(2) Soundness: for any common input *x* ∉ *L*, any interactive Turing machine *P*′, and polynomial *p*(·),
  (5) $$\begin{array}{c} \text{Pr}\left[V\left(x,R,P^{\prime }\left(x,R\right)\right)=1\right]<\frac{1}{P\left(\left|x\right|\right)}. \end{array}$$
(3) Zero knowledge: for any *x* ∈ *L*, there is a probabilistic polynomial time algorithm *M* such that
  (6) V(x)=(x,R∈{0,1}c(|x|),P(x,R))≈Mc(x)x∈L.

### 2.3. Witness Indistinguishability

**Definition 3 (witness indistinguishability [19])**

Let *L* be an NP language, let (*P*, *V*) be the interactive proof system of *L*, let *R*_*L*_ be the witness relation of *L*, and let *z* ∈ {0,1}* be the auxiliary input of *V*. (*P*, *V*) is said to be witness indistinguishable for *R*_*L*_, if, for any probabilistic polynomial time interactive Turing machine V* and any *ω*_1_, *ω*_2_ ∈ *R*_*L*_(*x*), the following probability ensembles are computationally indistinguishable:

(7) {(P(ω1),V∗(z))(x)}x∈Lz∈{0,1}∗  ≈{(P(ω2),V∗(z))(x)}cx∈Lz∈{0,1}∗.

Witness indistinguishability is a weaker notion of zero knowledge, but it is sufficient to ensure the security of cryptographic protocol in some applications. It is worth mentioning that witness indistinguishability is closed under concurrent composition.

## 3. Research Progress of NIZK

### 3.1. The Definition and Models of NIZK

In view of the important theoretical and applied value of zero knowledge interactive proof system in the fields of computational complexity and cryptography, its inherent nature and characteristics have caused much attention, such as interactivity and the randomness of participants and auxiliary input. Oren [20] first proves that NIZK proof systems only exist for BPP languages in the plain model (without any trusted set-up assumption). In 1988, NIZK proof system based on the CRS model is proposed by Blum et al. [2]. CRS is generated by a trusted party and is accessible to both the prover and verifier. This model requires only the randomness of CRS, not relying on its privacy, so CRS model is more practical than interactive model. In the same year, de Santis et al. [21] discuss NIZK in another model which is called NIZK with preprocessing. The idea of preprocessing model derives from one time pad [22]: in the preprocessing stage, the prover chooses an *n*-bit string *v* ∈ *V* and convinces the verifier (*v* ∈ *V*) through interactive zero knowledge proof; in the subsequent interactive stage, the prover constructs a proof of the statement to the verifier; then the verifier can verify the correctness of the statement according to *v* ∈ *V*. The disadvantage of preprocessing model is that the prover and verifier need to interact first, and the length of statement proved in the interactive stage is limited by the length of *ν*. In addition, preprocessing model is stronger than CRS model because the two parties can generate CRS in the preprocessing stage. Comparing the two models, CRS model is more reasonable, general, and practical. It is the widely accepted NIZK model now.

In 2004, Cramer and Damgård [23] proposed a secret key model of NIZK whose security depended not on CRS but assuming an appropriate secret key to exist between the prover and verifier. In 2007, Groth and Ostrovsky [24] put forward multistring NIZK model. They point out that CRS needs to be generated by a trusted third party in the single string model. However, it is difficult to find a suitable third party in practical applications. Therefore, it can be considered that the common reference string is generated by multiple parties as long as most of them are honest. Meanwhile, they also present the first NIZK proof system in the multistring model.

### 3.2. NIZK Proof Systems of NP Problems

In early literatures, researches on NIZK are mainly focusing on the existence and effective constructions of NIZK proof systems for NP languages.

Blum et al. propose the first bounded NIZK proof system in [2]; that is, for different statements the proof system has to use different CRSs and the length of the statement is controlled by the length of CRS. Later, Blum et al. [3] present a more general NIZK proof system for 3SAT on the basis of [2], which allows a prover to prove many statements with the same CRS. However, the above proof systems are constructed based on specific mathematical problems.

Feige et al. [25] present the first NIZK proof system for NP based on general assumptions, and the construction is based on one-way permutations or certified trapdoor permutations for a polynomial time prover [26]. At the same time, they also introduce a hiding bit model and use witness indistinguishability to turn bounded NIZK into general NIZK proof system which allows many provers to use the same random string to prove different statements. Lapidot and Shamir [27] give the first publicly verifiable NIZK assuming the existence of one-way permutations. References [3, 27] separately show NIZK proofs of 3SAT problem and HC problem based on different assumptions, respectively. Then NIZK proof systems for general NP problem can be obtained by Karp reduction, but this kind of constructions engages a very high level of complexity. Thereafter, Damgård [28] designs NIZK proof system for SAT problem, making the construction of NIZK for NP problem more direct. Simultaneously, he also gives noninteractive statistical zero knowledge argument of HC problem under the preprocessing model.

Bellare and Yung [29] point out that the trapdoor permutation used in NIZK proof system in [25] requires additional verification and puts forward the corresponding solution. Following the hiding bit method in [25], Kilian shows a NIZK proof system for SAT based on one-way permutations, and the number of hiding bits is *O*(*n* log⁡^*c*^ *nk*). Since then, Kilian improves the construction in [30], which reduces the number of hiding bits to *O*(*kn*log⁡ (*n*/*ε*)). de Santis et al. [31] discuss the length of CRS in NIZK and show a NIZK proof system for NP problem whose CRS length is Θ(*n* ^*e*^ + log⁡ (1/*s*)), in which *e* > 0 is constant and *s* is the reasonable error bound. Boyar et al. [32] study short NIZK proofs and construct a NIZK proof system with the length of *O*(*mk*(log⁡ *m* + *r*)), in which *m* is the number of gates in the circuit and *k* is the length of the commitment. Moreover, this paper shows a NIZK proof system with length of *O*(*m*(log⁡ *m* + *r*) + *rk*) in the RO model as well, and, in specific applications, NIZK with appropriate length can be obtained by simulating RO.

### 3.3. NISZK and NIPZK

Statistical zero knowledge [1] plays a significant role in both practical application and theoretical study, because it reflects the inherent characteristics of zero knowledge and does not need to be constructed under cryptographic assumptions as computational zero knowledge. The existing results show that there is computational zero knowledge proof [33] system for any PSPACE language, and, for SZK, we have SZK *⊆* AM *∩* coAM [34, 35]. (Here we use SZK to denote “statistical zero knowledge,” while we use SZK to denote the class of languages which have statistical zero knowledge proof systems. NISZK and similar notions are defined, resp.) However, it is generally believed that NP *⊈* AM *∩* coAM; thus the studies of NISZK are only considered for specific non-NPC language.

Blum et al. [3] propose the first noninteractive perfect zero knowledge (NIPZK) proof system for quadratic nonresidue problem in coNP. Ostrovsky [36] proves that, for any nontrivial language, the existence of SZK and NISZK proof or argument system is a sufficient condition for the existence of one-way functions. Thereafter, de Santis et al. do some further researches on NISZK and NIPZK. First, they give a NIPZK proof for quadratic residue in [37] and a new method that turns noninteractive proofs into interactive proofs which can not only keep the same zero knowledge characteristics but also make the round of the converted interactive proof systems optimal. Then, they discuss the existence of PZK for quadratic nonresidue and the lower bound of CRS in the model with fixed CRS length in [38]. In 1998, de Santis et al. prove that NIZK is closed under complement by constructing a special language called “ID.” Since then, Goldreich et al. [39] study the relationship between SZK and NISZK and prove that NIZK is closed under Karp reductions as well as some other logical operations and ultimately conclude that SZK = NISZK. With the help of Boolean circuit composition theory, de Santis et al. [40] expand the scope of these two languages on the basis of the already known PZK and NIPZK. They point out that the languages got from specific language categories in NC^1^ circuit composition all have NIPZK. Besides, the idea also applies to SZK. Pass et al. [41] discuss NISZK in secret key model together with CRS model. They point out NIZK = NISZK = NIPZK = AM in the secret key model, while, in CRS model for nonadaptive definition, there is NISZK *⊆* AM *∩* coAM and, for adaptive definition, there is NISZK *⊂* BPP/1. Additionally, for the language undecidable by nonuniform polynomial circuits, the necessary and sufficient condition of NIZK is the existence of one-way function. Eventually they show an absolute result for the existence of NIZK: NIZK exists either for simple language only or for all AM languages.

The above results indicate that, for general NP language, noninteractive statistical (perfect) zero knowledge proof does not exist. Then, does noninteractive statistical (perfect) zero knowledge argument exist? Groth et al. [16] give an affirmative answer. They propose the first NIPZK argument system for language SAT and thus prove that there is a NIPZK argument system for any NP language. They also give the first adaptive UC secure NIZK argument. Afterwards, Abe and Fehr [42] put forward the first efficient NIZK argument system with adaptive soundness based on the KEA assumption, which also applies to any NP problem.

### 3.4. NIZK for Specific Problems

Since its invention, researches on NIZK are mainly focused on the theoretical problems. Although it is once used to construct CCA-2 secure encryption schemes by Naor and Yung [43] and signature schemes by Bellare and Goldwasser [44], these results are just theoretical feasibility without practical applications. One of the important reasons is that the construction of NIZK is not efficient. Early researches are mainly focused on NIZK proof systems for general NP problems, so the NPC problems such as SAT, 3SAT, HC, or G3C are usually taken for consideration. While, in practical applications, we instead consider certain types of problems (such as the computations in the bilinear group), therefore the NIZK proof systems for general NP problems have to be reduced to NIZK proof systems for specific problems, which greatly sacrifices the efficiency. How to construct efficient NIZK proof systems seems to be the key to promote their applications.

In 2008, Groth and Sahai [17] analyze the reasons why the past NIZK proofs are inefficient and put forward the famous GS proof framework that applies to all basic operations in bilinear group. NIZK proof system can be obtained simply and efficiently through instantiating GS proof according to different application backgrounds, which greatly simplifies the design of public key cryptographic algorithm and cryptographic protocol based on bilinear groups. Since then, Ghadafi et al. [45] revise and expand GS proof to make it applicable to more bilinear groups. Later, Groth [7–12] makes further improvements on some aspects such as the computational efficiency and length of NIZK. Besides, Damgård and Thorbek [46] show a NIZK proof system of integer multiplications.

### 3.5. NIZK and IZK

The relationship, comparison, and transformation between NIZK and IZK are also important research directions of zero knowledge proof systems. At first Blum et al. [2] point out that CRS model is weaker than interactive model; that is, NIZK proof system does not necessarily exist in language with IZK proofs. Then, is there a suitable model making NIZK and IZK equivalent?

In 2002, a new zero knowledge proof model known as the “HELP” model was proposed by Ben-Or and Gutfreund [47], in which a third party “Dealer” is assumed to exist. It is a probabilistic polynomial time algorithm for solving the common reference string. In 2007, Ciocan and Vadhan [48] prove that a language in AM has an interactive proof system only if there is a NIZK proof system in the HELP model. At the same time, they point out that this result applies to the computational and statistical zero knowledge, not relying on cryptographic assumptions. From then on, Chailloux et al. [49] prove that NIZK and IZK are equivalent in the HELP model. In 1990, Fiat and Shamir exhibit a method that transforms interactive protocol into noninteractive protocol, known as “Fiat-Shamir heuristic” in [50]. The method can be used to turn public-coin IZK proofs into NIZK arguments. But hash function is used in this transformation, so the NIZK argument can only be proved to be secure in the RO model. In 1994, de Santis et al. [37] present a new method that turns noninteractive proof systems into interactive proof systems, which can not only keep the same zero knowledge characteristics but also ensure the round complexity of the converted system to be optimal.

### 3.6. NIZK and Zap

In 2000, Dwork and Naor show a surprising result in [51]: there exists two-round public-coin witness indistinguishable proof system that does not use CRS. The authors call the proof system zap. In a zap, the verifier first sends a random string to the prover; then the prover replies with a message to complete the proof. Zaps have many applications such as the construction of concurrent zero knowledge, deniable authentication [51], and ring signature [52]. The paper also presents a construction of zap using NIZK and verifiable pseudorandom generator (VPRG).

As can be seen from the definition of zap, it has an important link with NIZK. In 2002, de Santis et al. discuss the length of random string in zap and NIZK proof of NP problem. They point out that if there is zap for NP problem, then the length of random string used will be Θ(*n* ^*e*^ + log⁡(1/*s*)) bits; if there is NIZK proof for NP problem, then the number of bits used will also be Θ(*n* ^*e*^ + log⁡(1/*s*)). In 2006, Groth et al. [15] propose a new method to construct NIZK proof as well as NIZK argument and give the first construction of noninteractive zaps.

## 4. The Applied Researches of NIZK

The inherent privacy and authentication properties of zero knowledge proof system make it widely used in the construction of cryptographic protocols. Generally speaking, IZK proof system is usually used to construct multiround interactive protocol in the plain model, for example, general two-party and multiparty secure computation, and mostly for designing protocols in an abstract way, while NIZK proof is usually integrated into the construction of specific, practical cryptographic algorithm and cryptographic protocols. This raises very high demands on the construction of efficient NIZK proof systems. At first, Blum et al. point out that NIZK can be used to design public key encryption schemes secure against chosen ciphertext attack. However, this paper only shows the possibility but does not give a specific construction. Since then, Naor and Yung [43] put forward the first CCA secure public key encryption scheme on the basis of probabilistic encryption [53] and NIZK. Bellare and Goldwasser [44] present a new method to construct signature and message authentication protocol with the help of NIZK. And the scheme obtained is secure against adaptive chosen message attack. In 1999, Sahai [54] extends the nonmalleability of cryptographic protocols to NIZK and proposes a method to transform general NIZK into NMNIZK. At the same time, this paper also gives an encryption scheme secure against adaptive chosen ciphertext attack.

On the other hand, NIZK is widely used in group signatures, ring signatures, and electronic voting. NIZK is first used to construct a provably secure group signature scheme in the standard model by Bellare et al. [55]. Thereafter Groth uses NIZK to construct a group signature with constant size as well as a completely anonymous group signature [6] scheme in the standard model. Zap is introduced to the construction of ring signature for the first time by Bender et al. [52]. Recently NIZK is used in shuffle verification by Groth et al. [4, 13, 14].

## 5. Summaries and Outlook

In the recent 20 years, researches on NIZK proof system and related theory have improved gradually. Recent research focuses are mainly concentrated on the application and efficiency improvement of NIZK proof system, including the following aspects.

1. Efficient NIZK proof and NIZK argument system that apply to specific application backgrounds: currently, the researches for NIZK efficiency are mainly concentrated on the computation in bilinear group, so it is worth deeply studying how to construct highly efficient NIZK protocol applicable to other mathematical backgrounds.
2. Other cryptographic tools that cooperate with the existing proof systems: recently, Abe et al. [56] propose structure-preserving commitments and signatures which apply perfectly to GS proof system so that it enables the modular design of the protocols and at the same time ensures the efficiency. At present, these researches are just beginning, and there are still a lot of problems in the efficiency and application of these schemes.
